# Exploring emerging psychopathological characteristics and challenges of novel depression subtypes: insights from the literature

**DOI:** 10.3389/fpsyt.2025.1613251

**Published:** 2025-07-02

**Authors:** Stefania Chiappini, Gaia Sampogna, Antonio Ventriglio, Giulia Menculini, Valerio Ricci, Mauro Pettorruso, Umberto Volpe, Giovanni Martinotti

**Affiliations:** ^1^ Department of Psychiatry, UniCamillus International University of Medical Sciences, Rome, Italy; ^2^ Department of Psychiatry, University of Campania “L. Vanvitelli”, Naples, Italy; ^3^ Department of Clinical and Experimental Medicine, University of Foggia, Foggia, Italy; ^4^ Section of Psychiatry, Department of Medicine and Surgery, University of Perugia, Perugia, Italy; ^5^ San Luigi Gonzaga Hospital, University of Turin, Orbassano, Italy; ^6^ Department of Neuroscience, Imaging and Clinical Sciences, G. D’Annunzio University, Chieti, Italy; ^7^ Section of Psychiatry, Department of Neurosciences/DIMSC, Università Politecnica Delle Marche, Ancona, Italy

**Keywords:** depression, modern-type-depression, clinical subtypes, digital depression, dual diagnosis, psychopathology

## Abstract

**Background:**

Depression is a widespread global health issue, significantly impacting all areas of life and is a leading cause of disability. Societal changes, including technological and cultural shifts, and the emergence of new psychoactive substances, have influenced how depression manifests, introducing new clinical dimensions and challenges in its understanding and treatment.

**Summary:**

This review summarizes from a psychopathological and clinical point of view the most important features related to novel depression subtypes, specifically: I) Early-onset depression; II) Depression and social disconnection; III) Depression and Alcohol/Substance Use Disorder; IV) Depression and Gender Dysphoria; V) Depression, stressful events, and other environmental factors. For each domain, the available research evidence is summarized, starting from theoretical contributions to the relevant psychopathological descriptors with special attention to issues relevant for the clinical practice.

**Key Messages:**

Overall, the phenomenology of depression is currently enriched by new symptomatology entities, including the dimensions of boredom, shame, fatigue, alexithymia, and emotional dysregulation. Those symptoms prevail in different novel subtypes of depression deserving in the clinical practice special attention and focused interventions.

## 1 Introduction

Depression is a significant global health issue affecting millions of people worldwide. According to the World Health Organization (WHO), approximately 3.8% of the global population suffers from depression, which includes about 5% of adults and 5.7% of those over 60 years old. This translates to roughly 280 million people worldwide dealing with depression​ (1, 2)​. The prevalence of depression varies by country. For example, currently, countries with some of the highest depression rates include Ukraine (6.3%), Australia (5.9%), and Brazil (5.8%)​. In the United States (US) around 8.3% of adults experienced at least one major depressive episode in 2021, with higher rates among young adults aged 18–25 at 18.6%​ (3)​. Depression affects women more frequently than men, with an estimated 6% of women globally experiencing depression compared to 4% of men​ (1)​. This gender disparity is also seen in specific populations, such as adolescents in the US, where 29.2% of females and 11.5% of males aged 12–17 reported experiencing major depression​ (3). Despite the availability of effective treatments for depression, a significant treatment gap exists, especially in low- and middle-income countries, where more than 75% of people with mental disorders receive no treatment. Barriers to care include insufficient investment in mental health services, a lack of trained healthcare providers, and social stigma​ (1, 2)​.

Depression is a multifaceted and debilitating condition that significantly impacts all aspects of life, including family and personal relationships, work performance, and overall health. It is a leading cause of morbidity and disability on a global scale, currently ranked as the fourth leading cause of disease burden. Depression is particularly prevalent in childhood and adolescence and is linked to harmful behaviors, e.g., substance abuse, increased suicide risk, poor academic performance, impaired social skills, and social withdrawal. The physiological, psychological, and social changes typically occurring during adolescence can elevate the risk for major depressive disorder and related depressive disorders (4). The phenomenology of depression encompasses a broad range of emotional, cognitive, and physical symptoms that profoundly affect an individual’s life. The changing dynamics of society, influenced by technological advancements, economic pressures, climate change and environmental concerns, as well as cultural shifts including changing family dynamics, have influenced the manifestation and understanding of depression, leading to the recognition of new forms and contributing factors (5). Emerging patterns of depression may be linked to specific personality disorders: abnormal clusters of personality characteristics are increasingly exacerbated and encouraged in this social context, as evidenced by the growing prevalence of personality disorder diagnoses and the consequent reduction in functioning associated with them (6, 7). The spread of these personality profiles, which could also be seen as a form of adaptation to a rapidly evolving social environment, appears to be predisposing and facilitating factors for the emergence of a depressive core. Moreover, they could also, in turn, alter its phenomenological presentation. These personality traits often align with specific characteristics such as heightened sensitivity to social evaluation, perfectionism, high neuroticism or low conscientiousness and an overemphasis on external validation, which are reinforced by cultural and technological shifts, including the pervasive influence of social media (8, 9). Additionally, the modern emphasis on individualism and performance may exacerbate vulnerabilities in individuals predisposed to self-critical or avoidant traits, potentially leading to a higher incidence of mood disorders (10). On this regard, Stanghellini (11) identified four archetypes of human existence, each characterized by distinct values and vulnerabilities that influence their depressive breakdowns: i) the *Homo melancholicus*, which demonstrates hyper-conformity and over-adjustment, with depressive crises rooted in a profound sense of loss; ii) the *Homo œconomicus*, whose values are utility and optimization, framing life as a cost-benefit equation. Its depressive state manifests as a sense of insolvency or failure to meet these productivity standards; iii) the *Homo dissipans*, which embraces excess and uninhibited expression, prioritizing transformative and often destructive behaviors; iv) the *Homo nevroticus* operating under strict limitations and prohibitions, living a life dominated by self-restraint: depression in this archetype is defined by frustration and demoralization. The emerging features and characteristics of depression in the context of socio-cultural and technological transformations could be understood as variations from a core depressive dimension influenced by the evolving socio-anthropological landscape, rather than entirely distinct from the core depressive dimension. The phenomenological approach adopted aims to capture these shifts without imposing rigid or definitive classifications. Depression is understood here as a multifaceted phenomenon, open to ongoing exploration and redefinition as new evidence and insights come to light. This perspective highlights the dynamic interaction between the fundamental depressive dimension and its contextual expressions, influenced by societal factors such as technological progress, economic challenges, and cultural evolution. Consequently, the framework presented in this paper should be viewed as a contribution to an ongoing dialogue rather than a comprehensive or final account. By acknowledging the impact of the current socio-historical context on depressive symptoms, we seek to deepen the understanding of how depression manifests in diverse settings, while allowing for future adaptations based on emerging evidence. From our standpoint, socio-cultural changes affect not only specific aspects of psychopathology but also the overall functioning of personality. We propose that recent shifts in affectivity (and consequently in depressive psychopathology) and behavior serve as significant indicators of broader transformations involving the entirety of personality functioning. This shift can be summarized in key points: postmodernity has partially eroded and altered our foundational matrices, fostering a sense of discontinuity in self-experience. This sense of discontinuity, in turn, has amplified identity diffusion, as reflected in the continuum “identity integration versus identity diffusion,” commonly used to assess levels of personality functioning. It is essential to recognize that the society we inhabit, dominated by technology, is founded on the acceleration of time, the contraction of spaces, and the loss of bodily transcendence as the basis for social order and economic profit.

## 2 Aim

Taking into account anthropological and sociological transformations recorded from the post-modern era up to current days, we have investigated in the literature the clinical characteristics of depressive disorders as they are currently described, bringing new symptoms to the forefront. In this review we explored the emerging psychopathological characteristics of what today is commonly diagnosed with the Diagnostic and Statistical Manual of Mental Disorders (DSM) definition of Major Depression and related disorder. The DSM concept of major depression is deeply rooted in the psychopathological contributions of Emil Kraepelin and Kurt Schneider. Kraepelin’s focus on cyclic mood disorders and biological underpinnings, combined with Schneider’s phenomenological insights into vital depression, provided the foundation for the symptom-based and episodic criteria used in the DSM today. These contributions reflect a synthesis of classical psychopathological traditions with the modern need for empirical precision and clinical applicability. This can be considered our starting point of comparison with regard of the concept of depression as intended today. In addition to describing the new depressive symptoms, we have tried to offer phenomenological-descriptive reflections, trying to capture the characteristics of the modern depressed patient with a psychopathological lens. These elements will inform the development of the concepts presented below and the methodology applied in this study (12–17).

## 3 Methods

The present paper is the outcome of two full-day meetings involving eight expert psychiatrists from diverse Italian academic and clinical backgrounds, all actively engaged in the clinical management of patients with depression. During these meetings, they shared their expertise on depression and its clinical features and treatments, analyzing the literature up to 2024, and discussed the evidence and unmet needs related. Authors were encouraged to share the state of the art in the phenomenology of depression, considering socio-cultural changes, technological advancements, economic pressures, climate change and environmental concerns. Specifically, questions were related to i) Depression in adolescents and young adults; ii) Depression and social disconnection; iii) Depression and Alcohol/Substance Use Disorder; iv) Depression and gender dysphoria; v) Depression, stressful events, and other environmental factors. An outline of the paper was developed, with each expert assigned to have a narrative review of the available evidence on a specific topic, integrating it with their clinical expertise. The manuscript, compiled from these contributions, was shared prior to the second meeting, where it was thoroughly discussed, and a group consensus was reached on each topic. The final version of the manuscript reflects the collective view of the expert group in an undivided and genuine manner.

## 4 Depression phenotypes: clinical and psychopathological characteristics

### 4.1 Early-onset depression: clinical features

The prevalence rates of major depressive disorder and dysthymia in adolescents are 8% and 4%, respectively. Over the past twenty years, the prevalence of depressive symptoms in adolescents has risen from 24% between 2001 and 2010 to 37% between 2011 and 2020. Overall, 34% of adolescents globally, aged 10–19 years, are at risk of developing clinical depression, which exceeds the reported estimates for individuals aged 18 to 25 years. To note, the prevalence and severity of depressive symptoms in adolescent populations increased after the emergence of the Coronavirus diseases 2019 (COVID-19) pandemic (18, 19), reaching a prevalence of 45% in 2021 (20).

Adolescence is a period of life with various emotional challenges, such as new academic or workplace pressures, increasing importance of peer and romantic relationships, reduced dependence on the family support, and changes in the system of values. These life changes normally coincide with an improvement in emotion regulation capacities (21). Indeed, in adolescence, the psychological processes of self-verification and self-enhancement play crucial roles in identity formation and development (22). Given increased independence and novel demands during adolescence relative to childhood, adolescents may have a particular need to regulate their emotions in response to stressors, and failure to do so may confer risk for mental health problems. Limited efficacy of internal regulatory strategies might be very common, shifting with age towards increased use of maladaptive strategies and decreased use of adaptive strategies, e.g., avoidance (of situations) and suppression of unwanted thoughts and feelings, which are linked with an increase in depressive and anxiety symptoms, and rumination, exacerbating depressive symptoms by reinforcing negative thinking patterns and emotional distress (23). Thus, heightened emotional reactivity, increased risk-taking, and impulsive behaviors may be characteristic of adolescence. This is coupled with an ongoing neurobiological development among circuitries implicated in the management of emotional processes (21). Current theories focus on maturation in activity and connectivity among the prefrontal cortex, striatum, and amygdala across adolescence, proposing that increasing prefrontal control over emotionally reactive subcortical regions enhances capacities to regulate negative emotions and manage impulsive tendencies (23). Stressful life events and childhood adversity are substantial risk factors for future psychopathology, and the capacity to regulate emotional reactions to these events may play a mediating role (24). This was largely demonstrated during the unprecedented emergency represented by the COVID-19 pandemic (25), but further events related to wars (26, 27), migrations (28) and issues related to climate change (29, 30) profoundly impacted the emergence of psychopathology in youth populations. Moreover, the progressive digitization of our society has led to an increased use of technology for daily life activities. While the digital transformation has improved quality of life by streamlining daily activities, it has also led to a rise in the pathological use of technological tools, contributing to the emergence of problematic Internet use and Internet addiction (31, 32). These epidemiological trends align with broader societal changes. For instance, the sharp increase in depressive symptoms between 2011 and 2020 coincides with the widespread adoption of smartphones and intensified social media use, which studies have linked to reduced psychological well-being (33). Additionally, the erosion of traditional family and ritual community support structures in post-modern society has left adolescents increasingly reliant on peer validation, exacerbating emotional instability (34). Consistently with this, the inner subjective experience of adolescents may vary across mental health conditions, such as mood disorders, psychotic disorders, anxiety, eating disorders, externalizing disorders, self-harm behaviors, etc. (35). To give an example, adolescents affected with a mood disorder may experience a change in one’s personal identity, or overwhelmingly intense emotions, the feeling of being trapped in the own mind or that the surrounding world is fading away. In the case of adolescents affected with psychotic disorders, they may experience a pervasive change in the lived world and self; and, similarly, for adolescents, self-harm behaviors can be a way to transform emotional or psychological pain into physical pain.

Reflecting on time and the body is essential for understanding the social and anthropological transformations of the adolescent depressed patient in the postmodern era. The construct of shame has emerged as one of the primary psychopathological organizers of new forms of depression. Whereas past depressive constellations were characterized by ‘guilt-inhibition-slowing-persecutory delusions,’ today’s constellations are more often marked by ‘dysphoria-anger-loneliness-emptiness,’ typical of borderline personality structures, and ‘emptiness-insufficiency-disappointment-shame,’ characteristic of narcissistic personality structures (36). As a result of these shifts, depressed patients now live in a chronic state of exposure to environmental influences, making them extremely sensitive and vulnerable, akin to an open wound that does not heal. In our fast-paced, profit-driven society, the fundamental experience of depression is changing, moving away from being primarily a response to loss and becoming more about deep-seated narcissistic shame, fueled by constant pressure to be “useful” and “productive. In this context, the instant takes on an infinite dimension where everything seems possible. Hyperconnectivity may be perceived as a form of omnipotent immediacy, yet it also leads to the loss of the body’s sanctity and transcendence, as it becomes enmeshed on the internet and trapped in direct, immediate communication (36, 37). This hyperconnected immediacy disrupts the continuity of temporality, severing the links between past, present, and future that are essential for narrative identity. Without this coherence, anxiety and shame fully expose individuals to the present: to the threatening abandonment by others, the painful presence of their devaluing gazes, and the scorn and disdain of social networks. However, these gazes are no longer real but are instead shaped by “likes” and comments disconnected from reality (38).

**Figure 1:**
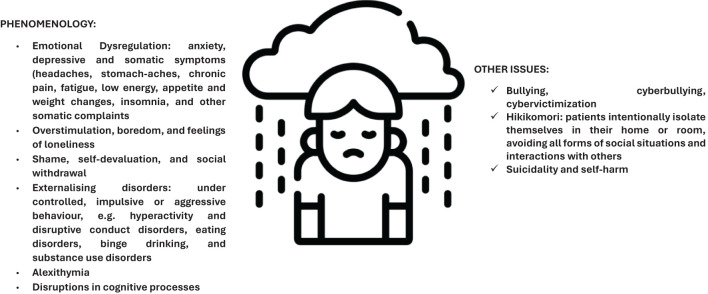
Early onset depression: clinical features.

The main clinical aspects of depression in young adults and adolescents are summarized as follows (**Figure 1**): i) *diminished expressivity*, manifesting as alogia and blunted affect. Alogia is characterized by a significant reduction in the amount or content of speech, leading to sparse and unelaborated conversations. Blunted affect involves a noticeable decrease in emotional expression, where individuals may seem emotionally flat, experiencing *reduced motivation and pleasure*, shown through symptoms like anhedonia, avolition, and asociality. Anhedonia is the inability to feel pleasure in activities that were once enjoyable, while avolition refers to a lack of motivation to initiate and sustain purposeful activities. Asociality is the apparent lack of interest in social interactions, leading to withdrawal and isolation from others. A second typical feature is related to the following aspects: ii) *emotional dysregulation* and conditions of anxiety, depression and somatic symptoms, often referring as *internalizing disorders*, involving negative emotions, such as sadness, worry, and fear, and physical symptoms, e.g. headaches, stomach-aches, chronic pain, fatigue, low energy, weight changes, insomnia, and other somatic complaints, leading to significant impairments in daily functioning (23, 38–42). iii) Despite the several benefits of social media, such as staying connected with friends and family, accessing support networks, finding communities of interest, etc., there are several concerns about their negative effects on adolescents’ mental health, including increased anxiety, depression, and feelings of anger, frustration, or inadequacy. Issues such as cyberbullying, social comparison, pressure to maintain a certain image contribute to negative self-esteem and stress; moreover, the constant connectivity and pressure to respond can lead to sleep disturbances and a lack of offline activities (43, 44). In this context, *digital depression* refers to a form of depression that is linked to excessive use of digital devices and online platforms (31). It has emerged with the widespread adoption of technology and the Internet, including the excessive amount of time using digital devices; the use of social media platforms with a constant exposure to curated images and posts of others’ seemingly perfect; negative online interactions, such as cyberbullying/cybervictimization, harassment, spreading rumors, or exclusion, which may have varying impacts on depression levels; the disruption of sleep patterns related to the blue light emitted by screens; feelings of isolation related to a digital communication able to connect people, but not to provide the same level of emotional support and connection as in-person relationships; feelings of anger whenever the possibility of prompt access to digital technologies is denied; and, finally, information overload (31, 45) and cyberchondria. To note, circadian changes in adolescence, including those related to higher use of social media as mentioned above, specifically interact with the intrinsic sleep-wake phase delay that occur in this period, leading to greater circadian instability possibly triggering the emergence of depressive symptoms (46). iv) *Boredom* is a foundational clinical element in new forms of depression, meant as a state of under-stimulation leading to the search for hyper-stimulation through online engagement and excitement. This emotional state, considered intrinsic to humans and possibly unique to Western culture, involves a simultaneous urge to act and a persistent longing (32). Clinically, it is characterized by chronic, unresolved tension, a lack of creative productivity, and disinterest in the external world, where everything appears static. The application of boredom to psychopathology is not a novel concept, with substantial research already dedicated to this area (32, 47–49). However, in the context of our study and its relevance to contemporary frameworks, boredom can be conceptualized as a subjective experience of a partial or complete lack of authenticity in interpersonal or contextual relationships (50). Lpidorou and Freeman highlight Heidegger’s idea of “profound boredom” as a state of existential confrontation, where one becomes aware of the emptiness or lack of meaning in life. This awareness can be transformative, leading to personal growth or deeper existential crises (51). It signifies an engagement with what is perceived as artificial, conformist, or emotionally distant. In this framework, reality becomes predictable, loses its sense of novelty, and is surrounded by a network of fixed symbols that hide its potential for change, energy, and exploration (51). Thus, time is perceived as an endless repetition of the present moment, with no hope of escape, emphasizing a semantic framework where the concept of boredom has significant temporal implications. Individuals experiencing boredom perceive time as a never-ending cycle of repetition, where aspects of continuity—such as similarity, stability, extension, and consistency—are diminished. The notion of progression or “becoming” is notably absent, making purposeful engagement with reality or establishing a particular interaction mode impossible, as described by Minkowski (52). Thus, the bored individual becomes passively trapped in a state of virtual waiting, effectively a prisoner of empty time. In this context, individuals with substance addiction or borderline tendencies toward self-harm seek to negate the emptiness of time, creating an illusion of synchronizing lived time with temporality and restoring a sense of unity in a pleasurable environment. Using the words of Kimura Bin and adapting them to the current anthropological dimension, this *intra-festum* temporality (8) reflects a point-like perception of time, where individuals immerse themselves in the present without a coherent sense of continuity. Instead of planning for the future, they define themselves by immediate experiences, which are often intense but lack depth and purpose. This present moment is passively experienced, not as a result of deliberate intentions and actions, lacking the richness deriving from integrating past experiences and anticipating the future. To fill this void, they seek fleeting pleasures and thrills, resulting in a disconnected series of momentary events rather than a coherent life narrative. Boredom, more than other states like apathy, vividly highlights the gap between an individual’s experienced time, directed toward the future, and the external world’s time. The temporal nature of boredom encapsulates a distressing temporal void, signifying a mismatch between subjective time and perceived external time. We can more appropriately describe instantaneity as the temporal framework of the postmodern condition. On one hand, it offers the illusion of a fast and direct connection with oneself and others, but on the other, it hinders the formation of strong bonds of belonging and the development of stable, coherent identities rooted in personal growth (53). This sense of instantaneous time is evident in the accelerated pace of modern technologies, the simultaneity of social interactions, and the fragmentation of time. These phenomena result in the decline of long-term narratives, replaced by disconnected fragments of stories (54). In the “now-life,” the urgency to act is no longer about acquiring and preserving, but rather about the constant cycle of discarding and replacing (55). The Internet, particularly portable devices, has brought an end to linear, sequential, slow, and cumulative communication that was once built through storytelling. Instead, today’s information is fragmented into billions of pieces, leaving individuals to reconstruct meaning for themselves—though many choose not to engage in this reconstruction at all. Differently from boredom, which lacks guilt and is marked by a sense of being dispersed in time, unable to place oneself within a historical context or establish a meaningful connection with the external world, in melancholy, time is stuck in the past, entangled with guilt and ruin; slowness characterizes melancholic expressions, while bored individuals retreat into physicality, exhibited by behaviors like impulsive spending, promiscuity, binge eating, substance abuse, reckless driving, and violence. v) Lastly, with regard to the construct of *shame*, this is described as an intense, painful feeling of being inherently flawed and unworthy, often resulting from a sense of failure or judgment by others which, contributing to self-criticism and rumination (56). It acts as a mediating factor in the relationship between depression and addictive behaviors, the latter considered as a coping mechanism to alleviate the distress associated with both depression and shame (56). On this regard, shame might be implicated in vulnerable narcissism-addiction pathway (57); moreover, higher levels of narcissism have been associated with lower perceived social support and life satisfaction, both mediating the relationship between narcissism and depression (58). Specifically, individuals with high levels of narcissistic rivalry often exhibit antagonistic thoughts and maladaptive relationships, making it challenging for them to find satisfaction in life. Similarly, those high in narcissistic rivalry typically have dysfunctional interpersonal relationships, resulting in lower perceived social support (58). Shame, as a generating element of depressive symptomatology, signifies the inability to meet societal expectations, creating a dissonance between the actual self and the ideal self. The guilt experienced by melancholic individuals is internalized and absolutized, manifesting as a constant sense of loss. In contrast, shame self-regenerates, tends to be totalizing, and is sometimes externalized through outbursts of anger directed at others or oneself, often leading to self-harm behaviors (3, 59). Finally, shame, for a more in-depth understanding, represents a state that serves a crucial signaling function, acting as an emotional indicator of a disruption in one’s sense of identity, an affront to narcissistic equilibrium, or the perception of a gap between the self and the ideal self. The main point we wish to emphasize is that shame primarily belongs to the dimension of the self, where individuals feel inadequate due to a perceived flaw that is incompatible with their idealized self-image. This flaw can be interpreted as a betrayal of societal expectations, a phenomenon that is further intensified in the virtual context of recent times. Shame, therefore, plays a significant role in both the onset and maintenance of depression, contributing to the underlying mechanisms that reinforce and perpetuate depressive experiences (60, 61). vi)*Alexithymia* (41, 62) is characterized by difficulties in identifying and describing both positive and negative emotions, and externally orientated thinking style, which significantly correlate with symptoms of depression, anxiety, and stress, and are strong predictors of mental health issues and under-controlled, impulsive, or aggressive behavior; examples include hyperactivity, disruptive conduct disorders, eating disorders, binge drinking, and substance use disorders (SUD). Self-harm, such as self-cutting or ingesting harmful substances, that result in non-fatal outcomes with the intention of causing self-injury, is a significant predictor of eventual suicide (63). A recently growing phenomenon is a specific type of behavior known as non-suicidal self-injury (NSSI); this is defined as any deliberate destruction of one’s own bodily tissues, enacted for non-suicidal reasons that are not sanctioned by social and/or cultural norms (64). It is particularly typical among youngsters and can be related to social networking, problematic social media use or fear of missing out, and can co-occur and precede a suicidal attempt (65). vii) From 85% to 95% of those who die by suicide among adolescents and adults have a psychiatric illness, particularly depression (41). Suicide emerges as an extreme consequence of shame, where the body loses its symbolic function as a vehicle for communication. The experience of an acute shame crisis causes the young, depressed individual to withdraw inwardly, paralyzing them and rendering them unable to control the external reality or implement coping mechanisms. The only perceived solution becomes disappearance, attacking the body (66). Finally, viii) adolescents affected by depression and anxiety often experience disruptions in their cognitive trajectories, which can impact various aspects of their cognitive functioning, such as attentive capacity (e.g., decreased concentration, distractibility, difficulties in deploying attention away from emotional stimuli, working memory impairment); problem-solving abilities (e.g., reduced cognitive flexibility, pessimistic bias, avoidance behaviors); interpretation and abstract thinking; and cognitive processing and memory issues (41, 67, 68).

These clinical aspects discussed here are described as part of a depressive dimension that does not exclude the co-occurrence of personality disorders (borderline or narcissistic) or anxious manifestations.

### 4.2 Depression and social disconnection

The rapid evolution of social structures, economic conditions, and demographic patterns has significantly reshaped the contemporary society. Indeed, it is characterized by increased uncertainty and complexity, due to global economic shifts, technological advancements, and changing labor markets (69–71). The traditional linear pathways from education to stable employment and independent adulthood have become less predictable and more varied leading to more fragmented and prolonged transitions to adulthood (70). The increasing proportion of elderly individuals presents new challenges in terms of healthcare, social security, and intergenerational support (72). Meanwhile, lower birth rates lead to a shrinking younger population, which can affect workforce size and economic growth. These demographic shifts also influence social policies and the dynamics of care within families, further complicating the balance between professional and personal life. Indeed, the traditional concept of family is undergoing substantial modifications. There is a noticeable shift from extended to nuclear family structures (73), accompanied by evolving roles within the household. Parental roles, in particular, have become more diverse, with both parents often participating in the workforce and sharing childcare responsibilities more equitably. Finally, a profound change in work dynamics, driven by technological advancements and globalization, has been leading to increased job insecurity and altered career trajectories (74). Moreover, strong social networks buffer against stress and help individuals navigate life challenges. In contrast, disconnection deprives individuals of this protective factor, increasing vulnerability to depressive episodes. Together, these factors create a complex social environment that impacts mental health and well-being. Trajectories toward *hikikomori*-like social withdrawal in modern society are potentially facilitated (72, 73). Originally a Japanese term, *hikikomori* refers to those individuals who intentionally isolate themselves, avoiding all forms of social situations and interactions with others, including friends and family members (75) (**Figure 1**). This phenomenon has expanded to other cultural contexts and now serves as a framework to describe similar behaviors observed globally (76–78). Over 60 years ago, it was proposed that large cultural, environmental, economic and social changes may specifically shape psychopathology and may exert their influence first in some countries where such conditions are particularly prone to the emergence of “culture-bound syndromes” or where those could be seen more frequently (79). Although recent changes in contemporary classification systems may have partly neglected the concept, the value of the social and economic changes in affecting the presentation of various psychiatric syndromes has been recently re-discovered (80) and, in particular, Hikikomori has been proposed as one of the most prominent psychiatric “boundless syndrome” of the 21^st^ century (81). We refer to a *hikikomori-*like condition, to distinguish it from the true *hikikomori* condition, which is rooted in well-structured social premises. Thus, a *hikikomori*-like condition might appear in different psychiatric disorders, such as in depression, severe anxiety disorders or even schizophrenia with negative features. Depressed mood, decreased motivation and activity are major symptoms that may present in the form of withdrawal-like outcomes (82); social anxiety disorder is a high comorbid psychiatric disorder among persons with *hikikomori*; and, finally, *hikikomori* behavior may be a precursor to suicidal tendencies (75). Modern-Type Depression (MTD), recently introduced by Kato, may share a similar etiopathogenetic background rooted in certain characteristics of contemporary society. MTD exhibits some overlap with the DSM-defined subtype of atypical depression and is characterized by social withdrawal, fatigue, low mood, and lack of motivation. These symptoms are often reactive in nature and commonly linked to specific situational factors, such as workplace stress (e.g., job insecurity, high performance expectations), interpersonal conflicts, or social pressures, unlike classical depression (73, 83). The phenomenon of social disconnection with the separate domains of social isolation and loneliness (84) prompts significant reflections on the evolving clinical perspective of depression. Loneliness defined as a negative emotion, an emotion of absence, related with the discrepancy between desired and existing relations, can be either emotional or social (85, 86). Emotional loneliness is described as a subjective experience resulting from the absence of a close bonding with a person, whereas social loneliness reflects an objective lack of contacts and social networks (87). Loneliness and its associated negative emotions, while more prominent in the elderly and characteristic of that stage of life, are also believed to be significant during adolescence and young adulthood due to the numerous social transitions and challenges faced during this period (88–92) (25, 93), and there have been heightened concerns about its effects during the COVID-19 pandemic (94–96). Although loneliness is a core feature of MTD, in the past Fuchs (97) conceptualized depression as a disruption in the natural bodily interaction with others, and Ratcliffe (98) described depressed individuals as perceiving their social interactions deficient. Depressed individuals often feel social goods are absent, their agency diminished, and others incapable of understanding their experiences, leaving them disconnected from the shared social world. This emptiness or hollowness is central to the depressive experience in MTD. Social disconnectedness in depression is experienced by the body’s experiential saturation with lethargy, tiredness, heaviness, sadness, and hopelessness. Rather than perceiving the body as an object-like entity, these embodied experiences dominate, precluding the ability to establish affective connections with others, leaving cognitive and other forms of interaction potentially intact (99). The symptom of psychomotor inhibition is a frequent feature in a body that has lost its normal fluidity of movement, and is resistant to any impulse toward the outside. The arrest of temporal becoming, the drive to movement, is thus one of the main symptoms of MTD: the experience of a deanimated body reflects the arrest of vital impulse, the depressive mood reflects the impossibility of being moved by emotions, and the delusion of guilt is the crystallization on the plane of thought and the register of morality of this immobility, of the blockage of personal becoming, of falling behind oneself and the rhythm at which time is seen to flow in the surrounding world (100). It is now evident that the over the past century, psychopathology, particularly within phenomenological frameworks, has consistently emphasized the profound connection between the arrest of vital impulses, their bodily manifestations, and the experience of temporality. Depression, especially in its severe form, has been conceptualized as a disturbance of lived time (101, 102), characterized by a temporal experience in which the crucial events have already irreversibly occurred. In this pathology, time is experienced in the form of *post festum* (8), meaning the already occurred, where the present is an eternal repetition of the past and the future is seen as entirely impossible. Feelings of isolation, estrangement, distance from the world and other people are common experiences in people with depression highlighted in several phenomenological studies (103–105) and deeply rooted in the body. In our society the body no longer crystallizes in the past but dissolves into the network, expanding its connections and becoming hyperconnected in the virtual realm. In this hyperconnected reality, the boundaries of time collapse: the past and future disappear, leaving only a perpetual, fragmented present. This situation can be described as that stagnation of internal time proposed by Minkowski (47, 101) following Bergson’s ideas of the inhibition of becoming, the desynchronization between internal time and world time (102), wherein the individual struggles to align their internal sense of time with the rapidly evolving present.

In conclusion, social disconnection, characterized by loneliness, isolation, a reduced number of meaningful social interactions, and the loss of strong support networks, can contribute to the emergence of new forms of depression, as observed in conditions like hikikomori and modern-type depression syndrome. These emerging psychopathologies of depression are deeply rooted in the embodiment, manifesting as a sense of fatigue and psychomotor retardation. Furthermore, they are significantly shaped by a collapse in the experience of time, leaving individuals trapped in a perpetual and fragmented present with a temporal stagnation that manifests as inertia and existential despair. This stagnation aligns with the embodied symptoms, where the body’s physical state mirrors the perceived immobility of time.

### 4.3 Depression and alcohol/substance use disorder

**Figure 2:**
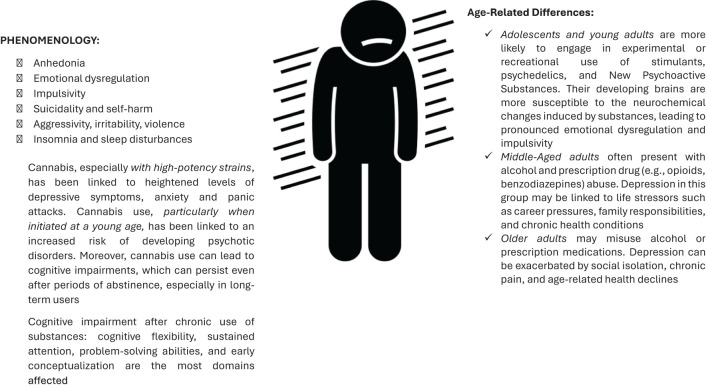
Depression and alcohol/substance use disorder.

The increasing prevalence of substances of abuse significantly contributes to the rise in psychopathological conditions, particularly the development of depressive disorders and psychosis. Besides conventional psychoactive substances, powerful new psychoactive substances (NPS), e.g. synthetic cannabinoids, synthetic cathinones, new synthetic opioids, etc. have become an emerging concern due to the rapidly evolving and virtually boundless online market they inhabit (103, 104). The relationship between depression and substance use is complex and bidirectional, with each condition potentially exacerbating the other (105, 106). Substance use can contribute to the onset or worsening of depression, while individuals with depression may turn to substances as a form of self-medication (107). Using traditional substances, particularly over long periods and in combination with other drugs, has a strong association with depression (104, 107, 108). Indeed, alcohol might initially be used to relieve depressive symptoms, but it ultimately worsens them by altering brain chemistry, causing dependency, and affecting social and occupational functioning​ (2, 3)​. Similarly, stimulant substances, such as cocaine and methamphetamine, can lead to severe mood swings and emotional dysregulation, with a high risk of anhedonia and severe depression during withdrawal or after prolonged use. These substances increase dopamine levels, which can lead to a temporary mood boost followed by a significant crash. Central Nervous System (CNS) depressants, including benzodiazepines and opioids, can lead to tolerance, dependence, and a cycle of using to avoid withdrawal symptoms, which often includes severe depressive states, anxiety or insomnia. Cannabis (108), especially with high-potency strains, has been linked to heightened levels of depressive symptoms, anxiety and panic attacks. These effects are more pronounced in individuals with pre-existing anxiety disorders sometimes using cannabis as a self-medication for their anxiety​ (109). Observational studies consistently show a link between cannabis use and an increased risk of psychosis, particularly schizophrenia. This risk is higher with earlier onset of cannabis use, more frequent use, and use of high-potency cannabis (high THC content) (110–112). Moreover, regular cannabis use can lead to cognitive impairments, affecting memory, attention, and executive functions. These cognitive deficits can persist even after periods of abstinence, especially in long-term users​ (113), defining clinical features specific for these patients Finally, NPS, although mimicking traditional drugs, are linked to increased risks of psychopathology due to their potent, unpredictable effects, and unregulated nature​ (104, 114)​. Indeed, several NPS have been directly or indirectly linked to severe adverse effects and fatalities, including suicide, e.g. new synthetic opioids have recently presented significant concerns, contributing to a rising number of overdose deaths in both the United States and Canada (115). The clinical presentation of depression with substance abuse in adolescents differs from classic psychopathological criteria, characterized by heterogeneous and evolving symptoms. These symptoms are sometimes masked by somatic complaints and complicated by high comorbidity with anxiety disorders, emotional dysregulation, personality disorders, medical illnesses, and frequent suicidality (**Figure 2**). Evidence suggests NPS use is a significant risk factor for impulsivity, violence and aggression in individuals with major mental disorders, and their use is highly reported in custodial settings as well (116–119).

In phenomenological terms, the proliferation of new substances of abuse has sparked significant debate regarding the temporal experience of these patients, underscoring the role of these substances in the genesis of new psychopathological manifestations (114). In this context, substances, particularly psychostimulants, intensify the patient’s experience of the present moment. Specifically, these substances have the capacity to fixate an infinite instant within a fragment of temporality. Messas (9, 120) refers to substance abuse as a condensation of reality, an intensification of the present moment that erases waiting time. This “continuous present” dominates the individual’s experience and disrupts the pre reflective experience of time. Consequently, the temporal horizon, which typically provides a backdrop for envisioning the future, contracts, causing the future to become both actualized and dissociated. The lived future, usually filled with aspirations and plans, loses its continuity with the present, leading to a narrowing of temporal distances and the collapse of all experiences into a singular, infinite present. To delve deeper into this concept, Messas introduces the notion of anthropological hyperpresentification (120) to describe the reduction of temporality to an exclusive focus on the present, severed from connections to both past and future within conscious awareness. Based on these observations, it is plausible to speculate that there is a dissociation of consciousness—specifically, a dissociation of the future from an unending present. Aspirations and plans become detached from the present, which expands and takes on an eternal quality, while the future loses its contextual framework, and aspirations lose their continuity with the present. This interpretation of the addict’s existence as entirely oriented toward the now is further supported by Ryan Kemp (121): drugs have no future beyond their immediate effects. As a result, the addict’s temporal consciousness becomes fossilized in the present due to the effects of the substance (120). The substance not only erases the future, but also uproots the past, hindering the depressed patient from narrating their life story and, most importantly, from constructing their own autobiographical narrative.

Key clinical features of these patients cannot be fully explained by or are not strictly limited to cases of complex depression, trauma, anxiety disorders, or personality disorders. They are summarized as follows (**Figure 2**): Anhedonia refers to a significant reduction in the ability to experience pleasure from activities that are typically enjoyable, often worsened by the brain’s altered reward pathways as a result of substance use. Deeply connected to this psychopathological dimension is emotional dysregulation, that exacerbates the impact of anhedonia, as the impaired ability to regulate emotions further disrupts the individual’s capacity to experience pleasure, creating a cycle that reinforces negative affective states and potentially contributes to the persistence of substance use as a maladaptive coping mechanism, leading to the onset and maintenance of psychopathological conditions, including depression and suicidal behavior (122–124). Indeed, biologically, emotional dysregulation involves dysfunctions in brain regions responsible for emotion processing and regulation, such as the prefrontal cortex and amygdala, crucial areas for executive functions, inhibitory control, and emotional responses. The prefrontal cortex, involved in planning and decision-making, when dysfunctional, results in poor impulse control and difficulty in managing stress, common in individuals with SUDs (125). Substance use can further impair emotional regulation by altering the brain’s reward and stress systems. Chronic drug use leads to neuroadaptations that reduce the brain’s ability to experience pleasure from natural rewards and increase sensitivity to stress. There is a positive linear correlation between loneliness, difficulty in emotional regulation, and drug abuse: indeed, each substance has the capability (in various ways) to create an internal sensation different from the real one, and substance use epitomizes a mechanism for avoiding and altering internal emotions, which cannot be managed and tolerated. ii)*Impulsivity* is characterized by increased risk-taking behavior and difficulty delaying gratification, often leads to substance use and self-destructive actions. This impulsivity is also embedded in the immediacy of temporality. Every event and change is experienced explosively, revealing a complete inability to distance oneself from events. This way of experiencing time can be understood as a defense mechanism in a psychodynamic sense (126). The temporal fragmentation underlying impulsive actions serves to mitigate the ambiguity and uncertainty of human relationships, which are perceived as threatening. iii) Individuals with SUD face a dramatically elevated risk of *suicide* compared to the general population. Research indicates that up to 40% of patients seeking treatment for substance dependence have a history of suicide attempts (127). Moreover, co-occurring mental health disorders significantly increase the likelihood of suicidal behavior. Conditions such as major depressive disorder, bipolar disorder, borderline personality disorder, and post-traumatic stress disorder (PTSD) can exacerbate feelings of hopelessness, impulsivity, and emotional pain, driving individuals toward suicidal actions (128). Stressful life events, including relationship disruptions, family conflicts, financial problems, and unemployment, prevalent among those with addiction, can trigger or worsen suicidal thoughts and behaviors, particularly when combined with the emotional instability associated with substance abuse (129). Finally, certain personality traits are frequently observed among individuals with substance dependence who exhibit suicidal behavior. Traits such as aggression, impulsivity, pessimism, and hopelessness can amplify the risk of suicide. Different substances have varying impacts on suicidal risk, e.g. alcohol use disorders are strongly linked to increased suicide risk, especially during periods of acute intoxication (130). Alcohol impairs judgment, reduces inhibitions, and increases impulsivity, making suicidal thoughts more likely to translate into actions. Chronic alcohol use can also deepen feelings of hopelessness and despair, further fueling suicidal ideation (130, 131). Individuals dependent on opioids, such as heroin, face a high risk of suicide due to factors like family history of suicide, childhood trauma, and concurrent psychiatric disorders. The intense withdrawal symptoms associated with opioid dependence can also drive individuals to suicidal actions as a means of escaping physical and emotional pain (130, 132). The stimulating effects of cocaine can exacerbate feelings of anxiety and paranoia, while the subsequent crashes can lead to severe depression. The combination of these mood swings and the impulsivity associated with cocaine use creates a volatile environment for suicidal behavior (130). Finally, individuals who abuse multiple substances simultaneously are at an even higher risk of suicide. The interplay of different drugs can lead to unpredictable mood states and further complicate emotional regulation. Polysubstance use often indicates a more severe level of addiction, with more pronounced social and psychological problems, thus increasing the overall risk of suicidal behavior. iv)*Poor sleep habits* are prevalent among substance users. Insomnia and sleep disturbances, which can be a direct effect of substance use or a symptom of depression, leading to a vicious cycle of worsening both conditions (133). Chronic insomnia can exacerbate depressive symptoms and is a risk factor for developing depression. Insomnia and depression share common neurobiological pathways, including dysregulation of the hypothalamic-pituitary-adrenal (HPA) axis and alterations in neurotransmitter systems such as serotonin and dopamine. v) SUDs have profound effects on *cognitive functions*, impacting various domains of mental processes. Executive functioning is significantly impaired, leading to poor decision-making, increased impulsivity, and reduced cognitive flexibility and planning abilities (134–137). Memory deficits are common, with both short-term and long-term memory being adversely affected, as seen in chronic users of alcohol and cannabis who struggle with holding, manipulating, and forming new memories (137). Attention is also compromised, with individuals exhibiting difficulties in sustaining and selectively focusing attention, a problem exacerbated by the use of stimulants and alcohol (136). Additionally, slowed cognitive processing speeds, impaired visuospatial abilities, and diminished language skills are observed among those with SUDs (134, 136). These cognitive deficits are underpinned by neurobiological changes, including reductions in grey and white matter in critical brain regions. While some cognitive recovery is possible with sustained abstinence and targeted rehabilitation, the extent of recovery varies depending on the severity and duration of substance use. Overall, acute effects vary depending on the substance, dosage, and frequency of use (134–136). In fact, early-stage users might experience short-term memory loss, reduced attention span, and impaired judgment and decision-making. These effects are often reversible with cessation of use. With continued and chronic use, cognitive impairments become more pronounced and persistent.

### 4.4 Depression and gender dysphoria

According to the DSM-5, Gender Dysphoria is defined as a marked and persistent incongruence between an individual’s experienced gender and assigned sex, accompanied by clinically significant distress or impairment in social, occupational, or other important areas of functioning (138). For children, the diagnosis requires at least six of the following criteria over a minimum of six months: a strong desire to belong to another gender; preferences for clothing, play, roles, or companions typical of another gender; and a pronounced aversion to one’s anatomical sexual characteristics. For adolescents and adults, at least two criteria must be met, such as a strong desire to eliminate primary or secondary sexual characteristics or to possess those of another gender. This incongruence can be conceptualized through the framework of pre-reflective bodily awareness, as discussed by Gallagher and Zahavi, where individuals typically experience their body as an integrated part of their identity. In Gender Dysphoria, this alignment is disrupted, resulting in an acute perception of the body as an object rather than a subject (139). Neuroscientific research supports this understanding through the concept of “embodied simulation,” mediated by mirror neurons, which elucidates how individuals internalize societal gender norms, intensifying the misalignment between body, mind, and identity (140).

The process of gender identity formation can be understood as an “evolutionary transition,” encompassing awareness of masculine, feminine, or transgender traits that extend beyond biological sex to include social roles and body image (140). The concept of identity, or the self, has historically been framed within modern philosophical, phenomenological, and psychological discourse as a structured entity enabling individuals to recognize their continuity over time. The discourse on identity has long aimed to define the self’s characteristics to achieve a coherent understanding of its nature. Initially, the focus was on delineating the thinking self’s properties, with William James differentiating in a dualistic approach the “I,” the conscious subject capable of self-reflection, from the “Me,” the object of reflection. This differentiation is particularly relevant in contemporary discussions of gender identity, as it underscores the tension between the introspective self (the “I”) and the socially constructed aspects of identity (the “Me”). In the context of gender, the “I” represents an individual’s internal sense of self, while the “Me” aligns with how gender roles and expectations are shaped by external societal pressures. Although preserving the self’s unity integrating thoughts and bodily experiences into a unique personal identity, this does not fully explain the subject’s interaction with the environment and relationships (141), while, in social psychology, the concept of identity has been explored concerning others. Indeed, Cooley (142) introduced the “looking-glass self,” proposing that self-concept is shaped by others’ responses and evaluations. Mead (143) expanded on this by articulating the self as comprising the “I,” which remains unaltered by socialization, and the “Me,” which is shaped by internalizing societal attitudes. In contrast, Goffman (144) conceptualized individuals as enacting different social roles daily, reflecting a dual identity as both introspective actors and social characters. This perspective highlights how individuals may adapt their gender expressions depending on societal expectations, reinforcing the idea that identity, including gender, is both internally experienced and externally negotiated. Thus, the social contribution of others is essential for constructing a complete and structured self. Neisser (145) identified five distinct aspects of the self to address its complexity: the Ecological Self, Interpersonal Self, Extended Self, Conceptual Self, and Private Self. However, reducing individuality to a mere reproduction of societal influence does not reconcile the inconsistencies between our immediate awareness of identity and its formation through experience. Persson (146) redefined identity as encompassing three traits: being the percept of experiences, perceiving oneself as a subject, and recognizing lived experiences as one’s own. Despite of this, the concept remains critiqued for its difficulty in describing a transcendent entity with material attributes. Husserl (147) introduced the concept of *intersubjectivity*, a fundamental premise for constructing individuality and personal identity within experiential reality. Ricoeur (148) further distinguished between the “I” and the self, proposing the notion of narrative identity to reconcile the static and dynamic aspects of the self, including experiences of psychological and bodily change. On this regard, the centrality of the body in identity cannot be ignored; the body is not merely an object but the medium through which we present ourselves to others, thereby determining gender roles. Unlike other objects, the body is the center of every experience, an intentional subject (142).

The “evolutionary transition” from corporeal identity to gender identity involves an individual’s awareness of their masculine, feminine, or transgender characteristics, which are distinct from biological sex and encompass social roles and body image. This term, “evolutionary transition,” would be better understood as a psychological and social progression rather than a biological process, emphasizing the role of lived experiences and social interactions in the development of gender identity. Connell (149) argued that gender should be understood in relation to bodies and their actions, rather than being reduced to mere sexual anatomy. This perspective aligns with the view that gender identity is not only a reflection of physical characteristics but also emerges from the ways individuals enact and express their gender in everyday life. Connell argues that gender is best understood in terms of bodily actions and expressions rather than being reducible to sexual anatomy, emphasizing the social and performative nature of gender identity (149). This perspective is critical in understanding the experiences of transgender individuals, where the incongruence between physical embodiment and gender identity often manifests as a profound and ongoing negotiation rather than a static “disturbance.” The dominant framework of heterosexual monogamy imposes significant pressure on individuals whose gender roles or identities conflict with their physical appearance. Stoller (150) introduced the concept of core gender identity, which is linked to the perception of inhabiting a sexually characterized body and is thought to emerge early in childhood. While transvestism may involve temporarily adopting the opposite gender’s social role without posing a fundamental challenge to identity, transgender individuals experience a deeper and more profound misalignment involving corporeality and identity, irrespective of their sexual orientation. The term “disturbance” in this context could be reframed to capture the complex interplay of gender dysphoria, societal expectations, and personal identity. Specifically, transgender experiences might be more accurately described as an ongoing negotiation to reconcile a misalignment between bodily self-awareness and gender identity, rather than being reductively framed as a disturbance.

This historical digression, centered on the narrative of the self, can serve as a key to understanding psychopathological conditions that may arise. In Western culture, the concept of gender has been deeply ingrained in our psyche as a foundational element—we must know: is it a boy or a girl? Susan McKenzie (151) (a Jungian analyst and scholar, notes that the first question asked at the moment of birth is, *“Is it a boy or a girl?”* reflects a model in which the child is expected to externally perform their gender in the construction of a social identity. Consequently, gender assignment remains a critical element for achieving cultural understanding. Therefore, constructing cohesive and socially credible narratives strengthens the sense of authenticity in our “True Gender Self,” improving mental health and boosting self-esteem. Conversely, when our gender is not easily recognizable to others, it undermines our sense of identity, authenticity, and social status, leading to genuine depressive conditions (152). In recent years, the number of young patients diagnosed with gender dysphoria or/and gender-diverse identity—including non-binary and questioning sexual identities—has considerably increased (153). Epidemiological studies suggest that the prevalence of gender dysphoria is approximately 0.5% in the general population, with some variations depending on the country and specific demographic factors ​ (154, 155). High prevalence rates of depression (33.3%) and anxiety (29.6%) are identified among transgender people, and they are significantly associated with younger age, being unemployed, worse self-rated health, and having at least one chronic disease (156).

**Figure 3:**
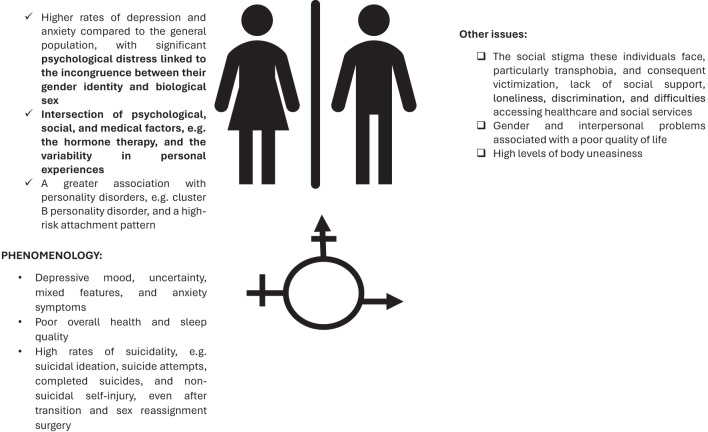
Depression and gender dysphoria.

Due to the multifaceted nature of the issue, arising from the intersection of psychological, social, and medical factors, e.g. the hormone therapy, and the variability in personal experiences, the understanding (and treatment) of depression in this population is very complex (157). Studies report higher rates of depression and anxiety compared to the general population, with significant psychological distress linked to the incongruence between their gender identity and biological sex. Specifically, higher rates of depression have been reported in gender dysphoric adolescents compared to their cisgender peers (153). Other studies emphasize that half of transgender youths are diagnosed with depression and anxiety disorders, as well as poorer overall health and sleep quality (158, 159). Addictive behaviors are also common in this population, representing a contributing factor that can complicate the clinical presentation (160). Similarly, increased rates of suicidality, e.g. suicidal ideation, suicide attempts, completed suicides, and NSSI have been reported (160, 161) (**Figure 3**) and remain significantly higher than in the cisgender population, despite gender-affirming medical interventions, such as sex reassignment surgery and hormone therapy (162, 163). These data may indicate a vulnerability to mental health problems in this particular category of subjects. Individuals experiencing this distress often find it challenging to align their gender expression with society’s conventional, rigid binary roles of male and female, leading to cultural stigmatization. This misalignment can contribute to relationship issues with family, peers, and friends, resulting in interpersonal conflicts, societal rejection, symptoms of depression and anxiety, substance abuse disorders, and a diminished sense of well-being (164). In fact, trans individuals were found to receive, or perceive themselves to receive, less social support from their family and friends compared to their non-trans siblings and the general population (163, 165). The social stigma these individuals face, particularly transphobia and consequent victimization, lack of social support, loneliness, discrimination, and difficulties accessing healthcare and social services, as well as gender and interpersonal problems (166), are associated with a poor quality of life in trans people (163, 167). Additionally, the high levels of body uneasiness observed in this specific group (168, 169), a greater association with personality disorders (170), and a high-risk attachment pattern (171) may contribute to their mental suffering.

In conclusion, the combination of various factors associated with a fragile and fragmented sense of self contributes to the development of depressive symptoms and an increased demand for support. Gender non-conformity often places individuals on the margins of societal acceptance, exposing them to exclusion, discrimination, and misrepresentation. This pervasive stigmatization cultivates a profound sense of isolation and diminished self-worth. At the root of this issue lies the dominance of cisnormativity—the societal expectation that gender identity must align with the sex assigned at birth. For transgender and gender-diverse (TGD) individuals, this rigid framework invalidates their lived experiences, leading to feelings of alienation and a lack of acceptance. From an early age, many TGD individuals grow up in environments where familial support is absent or conditional. Families that discourage authentic gender expression force children to suppress their “True Gender Self,” creating a psychological divide between their internal identity and the outward persona they are compelled to present. This suppression, beginning in formative years, often leads to chronic emotional distress and depression, shaping lifelong struggles with self-acceptance (172). The challenges extend into external environments, where bullying, violence, and misgendering are all too common, particularly in schools. These traumatic experiences leave deep emotional scars, often resulting in internalized transphobia and heightened anxiety. For many, the cumulative impact of these experiences evolves into persistent depressive states (173).

Institutional systems, especially within the medical sphere, can exacerbate this distress. The pathologization of gender diversity by medical frameworks creates additional barriers. The strict criteria often required to access gender-affirming treatments compel individuals to conform to restrictive narratives, undermining their authenticity and intensifying feelings of inadequacy. These challenges are further compounded by societal pressures and interpersonal rejection. TGD individuals frequently find it difficult to build meaningful social connections due to prejudice and misunderstanding. The expectation to “perform” gender in alignment with traditional norms amplifies feelings of loneliness and depression, creating a self-perpetuating cycle of alienation (174, 175).

At the heart of these struggles is a profound intrapersonal conflict. The dissonance between one’s physical body and gender identity can manifest as a significant existential challenge. This incongruence, intensified by societal emphasis on outward appearance, deepens self-doubt and magnifies depressive symptoms, making the journey toward mental health and well-being particularly arduous for TGD individuals.

### 4.5 Depression, stressful events, and other environmental factors

The role of stress on mental health and its role as possible trigger factor for mental health problems has been clearly introduced in mental health field by the development of the concept of neurasthenia. This concept has been introduced for the first time by Beard in 1869 (176), and evolved in the late 1950s when the Swiss psychiatrist Paul Kielholz (177) described “exhaustion- depression” as a condition characterized by prolonged psychosomatic symptoms due to sustained stress, particularly in businesspeople.

**Figure 4:**
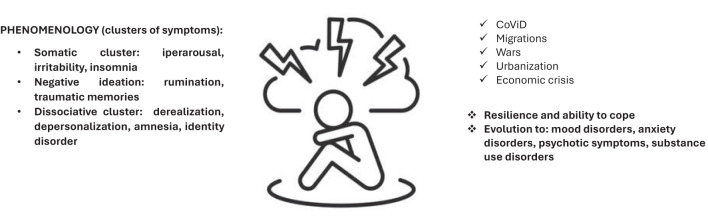
Depression and stressful events and other environmental factors.

Neurasthenia, as described by Beard (176), shared significant overlap with exhaustive depression, encompassing symptoms such as sadness, self-reported aches and pains, fatigue, elevated temperature, increased blood pressure, dyspepsia, and exaggerated motor reflexes. The condition was thought to result from the depletion of the CNS’s energy reserves, a consequence of modern civilization’s stressors, including urbanization and the competitive business environment. Indeed, it was predominantly associated with upper-class individuals and professionals in sedentary occupations. In the latest iteration of the classification system, ICD-11 (178), the concept of neurasthenia has been abandoned. However, modern-day conditions such as chronic fatigue syndrome and fibromyalgia show significant overlap with neurasthenia and exhaustive depression. These conditions worsen with stress, exertion, and weather changes, potentially representing forms of depression closely linked to the social changes of modernity and post-modernity. These include increased workplace competitiveness and workaholism, both associated with higher risks of depression, anxiety, and burn-out (**Figure 4**).

Stress can affect people in many different situations, and in particular it has been documented its detrimental impact in the workplace. In fact, due to a lack of support from supervisors and other colleagues or being involved in a working environment having little control over work processes represent significant risk factors for mental health and for the development of mental health problems. The relationship between levels of stress and working performance is bidirectional: perceived pressure can be useful to keep the individual alert, motivated, able to work, and learn, but, when it exceeds a certain threshold—which varies among individuals—becomes excessive or unmanageable, causing stress (179). The workplace represents a relevant source of stress for workers, being a risk factor for many mental disorders and psychological difficulties, including burn-out syndrome. Healthcare workers and other help-professions are particularly susceptible to work-related stress. Stress can affect workers in many different situations, and it is due to a lack of support from supervisors and other colleagues or to having little control over work processes. Stress can negatively influence employees’ health and their work performance. The workplace represents a relevant source of stress for workers due to excessive workloads, moral violence, work processes, interactions with patients’ families, professional and administrative demands, resource constraints, and lack of management support. The term “burnout” describes a physical and emotional strain specifically occurring in the work environment. Burnout syndrome is also known as chronic work-related stress syndrome (180).

Recently, the COVID-19 pandemic has represented an unprecedented traumatic event that has severely impacted social, economic, and health well-being worldwide. In Italy, the COvid Mental hEalth Trial - specifically designed to evaluate the impact of the COVID-19 pandemic and its containment measures on the mental health of the Italian general population – found that subjects with pre-existing mental health problems reported high levels of stress, anxiety and depressive symptoms as well as they were at higher risk of acute post-traumatic symptoms compared to the general population and health care professionals (181, 182). The levels of acute post-traumatic symptoms were higher in younger and female respondents. The COVID-19 pandemic has considerably influenced all domains of people’s lives worldwide, high increase in overall psychological distress and several clinical conditions (183, 184). The adoption of containment measures has contributed to the occurrence of several stress-related conditions and deterioration of pre-existing mental conditions (185, 186). The inclusion of the COVID-19 pandemic as a trauma-related factor builds on emerging research that underscores its unique role as a global event with long-lasting psychological impacts. Unlike localized traumas, the pandemic has created a shared global stressor, introducing novel challenges to mental health and exacerbating pre-existing vulnerabilities in diverse populations. This perspective highlights the pandemic’s role in reshaping our understanding of trauma-related disorders in a modern context.

Other cultural and social factors that should be considered as potential sources of stress include urbanization, globalization, pollution, climate change and lack of green space, just for quoting some social determinants of mental health (187–193). In particular, fast urbanization and the quality of urban neighborhoods, play a role in increasing the levels of anxiety symptoms and trauma-related symptoms (190–193).

Women living in urban areas, in particular, are more vulnerable to PTSD due to higher exposure to violence and crime (191, 194). Trauma-related symptoms can be grouped into somatic symptoms (e.g., emotional distress or physical reactions), negative changes in thinking and mood (e.g., flashbacks, nightmares, avoidance), and dissociative symptoms (e.g., emotional numbness, derealization). PTSD can evolve in various ways depending on individual differences, the nature of the trauma, and the availability of support systems. If symptoms persist beyond a month, a diagnosis of PTSD may be considered, with chronic PTSD potentially lasting for years and severely impacting daily functioning. Complex PTSD, recognized within the ICD-11 (178), arises from prolonged, repeated trauma and includes additional features such as affect dysregulation, interpersonal difficulties, and a negative self-concept (193). Migrants, due to their cumulative and chronic traumatic experiences, are particularly susceptible to complex PTSD, which often manifests with intense emotions, dissociation, and identity disturbances (195–197). The intersection of urban environments, gender vulnerability, and migration, sheds light on how modern societal dynamics contribute to mental health disparities. Women and migrants are disproportionately affected, emphasizing the need for targeted interventions and greater awareness of these interconnected factors. The relationship between trauma and the onset of psychosis has been well-documented, with growing evidence suggesting that individuals exposed to significant trauma, such as childhood abuse or war-related experiences, are at an increased risk of developing psychotic disorders later in life (198). Migrants, particularly those who have undergone traumatic experiences during migration, such as violence, persecution, or displacement, are also more vulnerable to mental health conditions, including psychosis. Social isolation, cultural dislocation, and socioeconomic disadvantages further exacerbate these risks (199–201). Post-migration living difficulties—such as language barriers, unemployment, unstable housing, family separation, discrimination, and restricted access to healthcare—constitute a significant burden on refugee and migrant mental health, independently of pre-migration trauma. Longitudinal research shows that decreases in post-migration living difficulties are associated with substantive improvements in depression and anxiety among severely traumatized refugees, even when post-traumatic stress symptoms remain unchanged, suggesting that psychosocial interventions targeting daily stressors can enhance therapeutic outcomes (202). Emotion dysregulation has been identified as a key mediator linking post-migration living difficulties to various psychological disorders—such as PTSD, depression, and anger—emphasizing the importance of integrating emotion-regulation strategies in interventions addressing post-migration stress (203). Moreover, recent work on complex PTSD in Afghan refugees illustrates how difficulties in language acquisition and communication function as central nodes within a network of post-migration living difficulties, often exacerbating psychopathological symptoms (203).

Trauma-related conditions within the broader contexts of migration and social isolation, show the cumulative nature of risk factors that contribute to severe mental health outcomes, including psychosis (204, 205). This perspective reinforce the importance of integrating trauma-informed approaches in clinical and public health strategies to address these complex challenges effectively.

## 5 Discussion

Depression is a widespread mental disorder and a major contributor to global morbidity and disability, affecting individuals of all ages, cultural backgrounds, and socioeconomic groups. As our understanding of depression continues to evolve, new subtypes have been recognized, shaped by dynamic socio-cultural and environmental factors. This review is anchored in the concept of a core depression, as described by classical psychopathology and defined in the DSM, which serves as a foundational framework. Over time, this framework has been influenced and reshaped by the socio-cultural contexts of modern society, resulting in the development of novel depressive subtypes and distinctive symptom patterns. The review try to examine the phenomenological and psychopathological aspects of these emerging subtypes, emphasizing how societal transformations—such as advancements in technology, shifts in work environments, changes in social connectivity, and evolving cultural norms—have profoundly affected the presentation and experience of depression. The relationship between these different manifestations of depression is dynamic and multifaceted. They often co-occur, with one factor exacerbating another in a complex web of influence. A holistic approach that considers all these potential interactions is essential for accurate diagnosis, comprehensive treatment, and effective prevention strategies for depression. A potential interplay between these kinds of depression should be considered, e.g., social disconnection (e.g., bullying, peer rejection, lack of close friendships) could present as a significant contributor to depression in adolescents and young adults (23, 24). Conversely, depression can lead to withdrawal and further social isolation, creating a vicious cycle. Adolescents and young adults may turn to alcohol or drugs as a coping mechanism for depressive symptoms. This self-medication often exacerbates depression in the long run and can lead to the development of AUD/SUD. On the contrary, substance abuse can induce or worsen depressive episodes. Similarly, for adolescents experiencing gender dysphoria, the distress and societal pressures associated with it can significantly increase the risk of depression (37, 38). This can be compounded by a lack of support, discrimination, and difficulty accessing gender-affirming care (153, 166). Finally, adolescents and young adults are particularly vulnerable to the impact of stressful life events (e.g., academic pressure, family conflict, romantic relationship issues, traumatic experiences) (29). These stressors can trigger depressive episodes, especially in those with pre-existing vulnerabilities. Environmental factors like poverty, exposure to violence, or lack of access to mental healthcare can also disproportionately affect this age group.

A key contribution of this work is the identification of novel symptomatology entities, such as boredom, shame, fatigue, alexithymia, and emotional dysregulation, which differ from classical presentations of depression. These symptoms are further compounded by emerging patterns of drug abuse and rapid societal changes. Central to this discussion is the concept of embodiment, as these new depressive states manifest through psychomotor retardation and fatigue, emphasizing the body as a primary site of suffering. This intersection of phenomenology and psychopathology provides a fresh perspective for interpreting modern depression.

The notion of time emerges as a pivotal framework in understanding these new depressive forms. Traditional perspectives often associate depression with rumination and a fixation on the past. However, contemporary depressive subtypes are increasingly characterized by a fragmented sense of time, wherein individuals become trapped in an unstructured, perpetual present. This “eternal now” disconnects individuals from the organizing frameworks of past and future, eroding their sense of purpose and direction. The accelerated rate of societal and technological advancements, occasionally influenced by substance use, intensifies the feeling of internal-external time desynchronization, making individuals feel alienated and profoundly “out of sync” with their surroundings. Furthermore, the collapse of future orientation, historically tied to a diminished sense of the future, is highlighted as a critical aspect of modern depressive experiences. Societal demands, such as hyperconnectivity and the pressures of instant gratification, intensify this collapse, undermining individuals’ ability to project themselves forward in time and envision meaningful goals.

Clinicians must remain informed about these developments to deliver culturally sensitive and evidence-based interventions. By tailoring treatments to address specific needs—considering cultural background, socioeconomic status, and unique stressors—healthcare providers can achieve earlier detection and better outcomes. In the era of personalized medicine, these insights are invaluable for selecting appropriate therapies, ranging from pharmacological interventions and psychotherapy modalities to holistic approaches incorporating lifestyle modifications and social support. Addressing one aspect of depression often requires simultaneously addressing the interacting factors to achieve lasting positive outcomes (37, 206). Interventions such as mindfulness practices, narrative therapy, and strategies that help individuals reconnect with meaningful future goals offer promising approaches to restructure the experience of time. Beyond individual treatment, these insights have broader implications for public health initiatives, enabling the development of programs to alleviate the societal burden of depression.
